# The effect of tumour necrosis factor-α (TNF-α) muteins on human neutrophils *in vitro*

**DOI:** 10.1155/S0962935193000055

**Published:** 1993

**Authors:** H. Tchorzewski, K. Zeman, J. Kantorski, E. Paleolog, M. Kahan, M. Feldmann, M. Kwinkowski, P. Guga, B. Szymanska, P. Parniewski, A. Wilk, J. Jarosz

**Affiliations:** 1 Department of Pathophysiology and Immunology Institute of Basic Medical Sciences Military Medical Academy Lodz Poland; 2 Kennedy Institute of Rheumatology Sunley Division London UK; 3 Virology and Microbiology Centre Polish Academy of Sciences Lodz Poland; 4 Department of Bioorganic Chemistry Polish Academy of Sciences Lodz Poland

## Abstract

Tumour necrosis factor-α (TNF-α) has been implicated as an important inflammatory mediator. *In vitro*, TNF-α is reported to activate human polymorphonuclear neutrophils (PMN), inducing responses such as phagocytic activity, degranulation and oxidative metabolism. Biological responses to TNF-α are initiated by its binding to specific cell surface receptors, and various studies have shown that the major TNF receptor species on PMN is the 75 kDa receptor. To verify the suggestion that the receptor binding domain includes the region close to the N-terminus of the TNF-α molecule, four TNF-α derivatives termed muteins were constructed, using a synthetic cDNA fragment substituting the N-terminal 3–7 selected hydrophilic or hydrophobic amino acids in the original TNF-α genomic DNA. Binding of muteins to PMN was assessed using monoclonal antibodies recognizing either the 55 kDa (p55) or the 75 kDa (p75) TNF receptor subtypes. Blocking by muteins of anti-p75 antibody binding to PMN was as expected from their N-terminal amino acid composition and hydrophilic properties. Hydrophilic muteins competed well with anti-TNF receptor antibodies for binding to the p75 receptor. In contrast, hydrophobic muteins were unable to block anti-p75 binding. Similarly, degranulation, chemiluminescence or enhancement of the PMN response to specific stimuli by the muteins correlated with the hydrophilic properties of the muteins. The significance of these observations in relation to the molecular structure of TNF-α is discussed.


Mediators of Inflammation, 2, 41–48 (1993)

### The effect of tumour necrosis factor-α (TNF-α) muteins on human neutrophils *in vitro*

H. Tchorzewski,[1,3,CA] K. Zeman,[1] J. Kantorski,[1] E. Paleolog,[2] M. Kahan,[2] M. Feldmann,[2] M. Kwinkowski,[4] P. Guga,[4] B. Szymanska,[4] P. Parniewski,[4] A. Wilk[4] and J. Jarosz[4]

[1] Department of Pathophysiology and Immunology, Institute of Basic Medical Sciences, Military Medical Academy, Lodz, Poland; [2] Kennedy Institute of Rheumatology, Sunley Division, London, UK; [3] Virology and Microbiology Centre, Polish Academy of Sciences, Lodz, Poland; [4] Department of Bioorganic Chemistry, Polish Academy of Sciences, Lodz, Poland

[CA] Corresponding Author

**Tumour necrosis factor-α (TNF-α) has been implicated as an important inflammatory mediator. *In vitro*, TNF-α is reported to activate human polymorphonuclear neutrophils (PMN), inducing responses such as phagocytic activity, degranulation and oxidative metabolism. Biological responses to TNF-α are initiated by its binding to specific cell surface receptors, and various studies have shown that the major TNF receptor species on PMN is the 75 kDa receptor. To verify the suggestion that the receptor binding domain includes the region close to the N-terminus of the TNF-α molecule, four TNF-α derivatives termed muteins were constructed, using a synthetic cDNA fragment substituting the N-terminal 3–7 selected hydrophilic or hydrophobic amino acids in the original TNF-α genomic DNA. Binding of muteins to PMN was assessed using monoclonal antibodies recognizing either the 55 kDa (p55) or the 75 kDa (p75) TNF receptor subtypes. Blocking by muteins of anti-p75 antibody binding to PMN was as expected from their N-terminal amino acid composition and hydrophilic properties. Hydrophilic muteins competed well with anti-TNF receptor antibodies for binding to the p75 receptor. In contrast, hydrophobic muteins were unable to block anti-p75 binding. Similarly, degranulation, chemiluminescence or enhancement of the PMN response to specific stimuli by the muteins correlated with the hydrophilic properties of the muteins. The significance of these observations in relation to the molecular structure of TNF-α is discussed.**

**Key words:** Inflammation, Muteins, Neutrophils, Receptor recognition, TNF

### Introduction

The multifunctional cytokine tumour necrosis factor-α (TNF-α) plays a role in the regulation of many biological responses *in vivo*, and has been implicated in a wide range of pathological conditions, including the host response to Gram-negative sepsis, cachexia and the acute phase response to infection and trauma.[1] During the course of an inflammatory response, TNF-α is released by monocytes, macrophages, T-lymphocytes and polymorphonuclear neutrophils (PMN). *In vitro*, TNF-α exerts a wide range of effects on target cells.[2,3] The first step in the induction of these various cellular responses is the binding of TNF-α to specific cell surface receptors. Two such receptors, binding both TNF-α and TNF-β, have recently been cloned by a number of groups, and the availability of specific monoclonal antibodies to these receptors has allowed the investigation of their distribution and regulation.[4–9] These distinct receptors, termed p55 and p75, are expressed in various amounts on different cell lines.[10,11]

The activation of PMN by TNF-α is a critical component of the inflammatory response, and *in vitro* TNF-α has been shown to enhance phagocytosis, oxidative metabolism and aggregation of PMN, as well as increasing cell surface expression of integrin molecules (CD11/CD18) and hence promoting adherence of PMN to vascular endothelial cells and transendothelial migration.[12–15] The existence on PMN of high affinity receptors for TNF-α has been demonstrated in several studies.[16,17] Based on their susceptibility to inhibition of TNF-α binding by anti-p55 and anti-p75 monoclonal antibodies, PMN are believed to display similar amounts of the two TNF receptor sub-types on their surface.[5,18,19] However, a study from the authors' laboratory demonstrated binding to PMN of anti-p75 TNF receptor monoclonal antibody alone, as measured by flow cytometry.[20] It was observed that binding of this antibody to PMN was markedly decreased by treatment of PMN with activating agents such as granulocyte macrophage colony-stimulating factor and formyl-methionyl-leucyl-phenylalanine, though not with interferon-γ, the tumour promoting phorbol ester PMA or calcium ionophore A23187.

The TNF-α domain responsible for receptor binding has been shown to be located in the

© 1993 Rapid Communications of Oxford Ltd

N-terminal portion of the molecule,[21,22] but the exact structure responsible for binding has not been precisely defined. For this purpose, four TNF-α derivatives termed 'muteins' were produced, in which 3–7 amino acids of native TNF-α have been replaced, using synthetic cDNA expressed in *Escherichia coli*. The authors have previously demonstrated differential binding of these muteins to Jijoye Burkitt lymphoma cells, which express only the p75 TNF receptor, and to the human epithelioid carcinoma HeLa cell line, which expresses the p55 receptor alone, suggesting that N-terminal amino acids play an important role in the binding of TNF-α to its cell surface receptors.[23] In the present study the ability of these muteins to prevent binding of anti-TNF receptor antibodies to PMN was examined. Additionally, the activation of PMN by muteins and native TNF-α was compared, and we report that the activities of the muteins on PMN correlate with their N-terminal amino acid composition and hydrophilic properties. These findings emphasize the importance of the N-terminus of the TNF-α molecule in binding to the p75 TNF receptor on PMN.

### Materials and Methods

*Materials:* Muteins III, IV, V and VI (for details of structures see Results) were constructed in the Department of Bioorganic Chemistry, Polish Academy of Sciences, Lodz, Poland, using synthetic oligonucleotides to induce changes in the cDNA encoding the 7 N-terminal amino acids of native TNF-α.[24] The cDNA was expressed in *E. coli*, and resulting muteins were purified by ion exchange chromatography. Amino acid sequences were analysed by automated Edman degradation using an Applied Biosystems ABI 477A protein sequencer. Mutein IV molecules formed inclusion bodies when expressed in *E. coli*, which were solubilized with 6 M guanidine HCl, and purified by phenyl Sepharose chromatography, before submission to sequence analysis. Endotoxin contamination amounted to approximately 1.9 ng endotoxin per mg protein, as estimated using a commercially available assay (Sigma Chemical Co., St Louis, MI, USA).

Recombinant human TNF-α (specific activity $5 \times 10^7$ U/mg) was supplied by Genentech Corporation (San Francisco, CA, USA) and by the Department of Bioorganic Chemistry, Lodz, Poland. Zymosan A, luminol, formyl-methionyl-leucyl-phenylalanine (fMLP) and Triton-X 100 were purchased from Serva (Feinbiochimica GmbH and Co., Heidelberg, Germany) and from Sigma Chemical Co. (St Louis, MI, USA). Biotinylated goat anti-mouse IgG and streptavidin–phycoerythrin were from Southern Biotechnology (Birmingham, AL, USA). The monoclonal antibodies used to detect the p55 (Htr-9) and the p75 (Utr-1) TNF receptors were kindly provided by Dr Manfred Brockhaus (Roche, Basel, Switzerland), and OX-20 (mouse and anti-rat $IgG_1$), which served as a control antibody, was kindly provided by Dr Don Mason (Oxford, UK). Gradisol G was obtained from Polfa (Kutno, Poland). Medium RPMI-1640, Hank's buffered salt solution (HBSS), phosphate buffered saline (PBS) and foetal calf serum (FCS) were all purchased from Gibco Laboratories (Paisley, UK). Normal human serum was prepared from blood collected from laboratory personnel.

*Preparation of peripheral blood neutrophils:* Whole blood anticoagulated with 10 U/ml heparin was obtained from healthy adult volunteers. PMN were isolated by a previously described method,[25] using rapid one-step centrifugation with Gradisol G (1.115 g/$cm^3$, 440 mOsm/kg $H_2O$). Cells from interphase were washed once in PBS, and resuspended in HBSS at a concentration of $5 \times 10^6$/ml. The neutrophils obtained were >96% pure and viability was greater than 98%, as determined by Trypan blue exclusion.

*Immunofluorescence studies:* PMN ($0.5 \times 10^6$/ml) were suspended in HBSS supplemented with 10% (v/v) normal human serum and incubated for 15 min at 4°C. The serum blocks cell surface Fc receptors, and hereby eliminates non-specific Fc-dependent binding. The PMN were incubated with either TNF-α or muteins, at a concentration of 100 ng/ml, for 30 min at room temperature, washed with PBS supplemented with 3% bovine serum albumin and 0.02% sodium azide, and incubated with optimal concentrations (10 μg/ml) of anti-TNF receptor monoclonal antibodies for a further 30 min at 4°C. In the initial set of experiments anti-TNF receptor monoclonal antibodies Utr-1 and Htr-9, which recognize p75 and p55 TNF receptors respectively,[26] were used. However, because only the p75 TNF receptor was visualized by flow cytometry on both resting and stimulated PMN, subsequent analyses were performed using monoclonal antibody Utr-1 only. PMN were then washed again, and incubated with 1:100 dilution (1 μg/ml) of biotinylated goat anti-mouse IgG, followed by 1:100 dilution (1 μg/ml) of streptavidin conjugated to phycoerythrin, both for 30 min at 4°C. The cells were washed twice more and analysed immediately using a FACStar flow cytometer (Becton Dickinson, CA, USA). TNF receptors were analysed on viable cells by gating on forward (FSC) and side scatter (SSC). The results were analysed using the Becton Dickinson LYSYS data analysis software package, and data are presented in the form of FACS profiles.

*Measurement of enzyme secretion:* PMN were resuspended in PBS at a concentration of $2 \times 10^6$/ml. In some experiments PMN were pretreated with 5 μg/ml cytochalasin B for 15 min at 37°C before use. The appropriate concentrations of either TNF-α or muteins were then added for a further 45 min at 37°C. Released enzyme activities were measured in cell-free supernatants. The activity of β-glucuronidase was evaluated using 0.01 M phenolphthalein glucuronate as a substrate.[27] Lysozyme activity was measured by a turbidometric method,[28] using egg-white lysozyme as a standard. Release of lactate dehydrogenase (LDH) from PMN was determined spectrophotometrically, using a commercially available assay from Sigma Chemical Co. (St Louis, MI, USA). This assay is based on the characteristic light absorption at 340 nm by diphosphopyridine nucleotide hydrogenase reduced coenzyme. All tested substances were diluted in PBS, which also served as a control for spontaneous degranulation of PMN. The enzyme activities released into the neutrophil supernatants were expressed as a percentage of the total activity released from PMN by treatment with 0.2% Triton-X 100.

*Chemiluminescence studies:* PMN were incubated for 30 min at room temperature in the presence of either TNF-α or muteins (1–100 ng/ml). Alternatively, PMN were pre-incubated for 30 min at room temperature in the presence of either TNF-α or muteins (1–100 ng/ml), before addition of fMLP ($10^{-6}$ M)). For the measurement of chemiluminescence (CL) generation, each sample contained 350 μl cells ($3.5 \times 10^5$), 20 μl luminol solution and 10 μl of either TNF-α or mutein. PBS was added to give a final volume of 1.0 ml.[29] CL generation was evaluated using a Luminometer 1251 (BioOrbit Turku, Finland) coupled to an IBM-PC At computer. This allowed the simultaneous examination of 25 samples. Investigations were carried out at 37°C and each measurement was made on three different PMN samples in triplicate.

### Results

*Structure of TNF-α muteins:* The N-terminal amino acids in the muteins, as compared to native TNF-α, are as follows:

| | | |
|---|---|---|
| Native TNF-α | Met-Val-Arg-Ser-Ser-Ser-Arg-Thr- | (hydrophilic) |
| Mutein III | Met-Lys-His-Lys-Arg-His-Arg-His- | (hydrophilic) |
| Mutein IV | Met-Phe-Met-Ala-Phe-Phe-Met-Met- | (hydrophobic) |
| Mutein V | Met-Val-Arg-Ser-Ser-Ile-Val-Ile- | (hydrophilic) |
| Mutein VI | Met-Arg-Ile-Arg-Met- | (hydrophobic) |

The hydrophobic and hydrophilic character of the muteins was evaluated according to the generally accepted algorithm.[30] The hydrophilic properties of mutein V were thus calculated to be slightly lower than those of either mutein III or native TNF-α.

*Immunofluorescence staining of PMN for TNF receptors:* This study confirmed the authors' earlier observations that PMN do not bind the anti-p55 TNF receptor monoclonal antibody Htr-9, as measured on a Becton Dickinson FACStar flow cytometer. In contrast, significant staining of PMN with monoclonal antibody Utr-1, specific for the p75 TNF receptor (Figure 1) was obtained. Pretreatment of PMN with human recombinant TNF-α (100 ng/ml) for 30 min completely abolished binding of Utr-1 to PMN, indicating that this monoclonal antibody binds to a surface site specific for TNF-α (Figure 2). Binding of Utr-1 was also blocked by similar pretreatment of PMN with mutein III, and to a lesser extent following pre-incubation of PMN with mutein V. However, treatment of PMN with muteins IV and VI had no significant effect on anti-p75 antibody binding (Figure 2).

*Chemiluminescence activity:* Treatment of human PMN with TNF-α results in the production of increased amounts of superoxide anions in response to a subsequent challenge with fMLP, reflecting release of myeloperoxidase from neutrophil azurophil granules, which can be measured as luminol enhanced chemiluminescence. From Figure 3, it is clear that when added alone, TNF-α and muteins III and V slightly stimulated superoxide generation by PMN, whereas muteins IV and VI possess only negligible PMN stimulatory activity. In contrast, treatment of PMN with fMLP ($10^{-6}$ M) induced significant generation of chemiluminescence, which was markedly enhanced by pre-incubation of PMN with either TNF-α, mutein III or mutein V prior

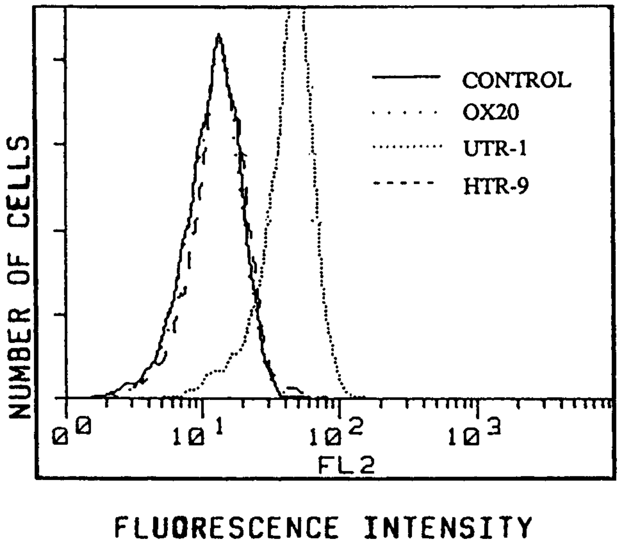

FIG. 1. Flow cytometric analysis of unstimulated human PMN stained with 10 μg/ml of monoclonal anti-TNF receptor antibodies Utr-1 and Htr-9, or with irrelevant control antibody OX-20. PMN bind anti-p75 TNF receptor antibody only.

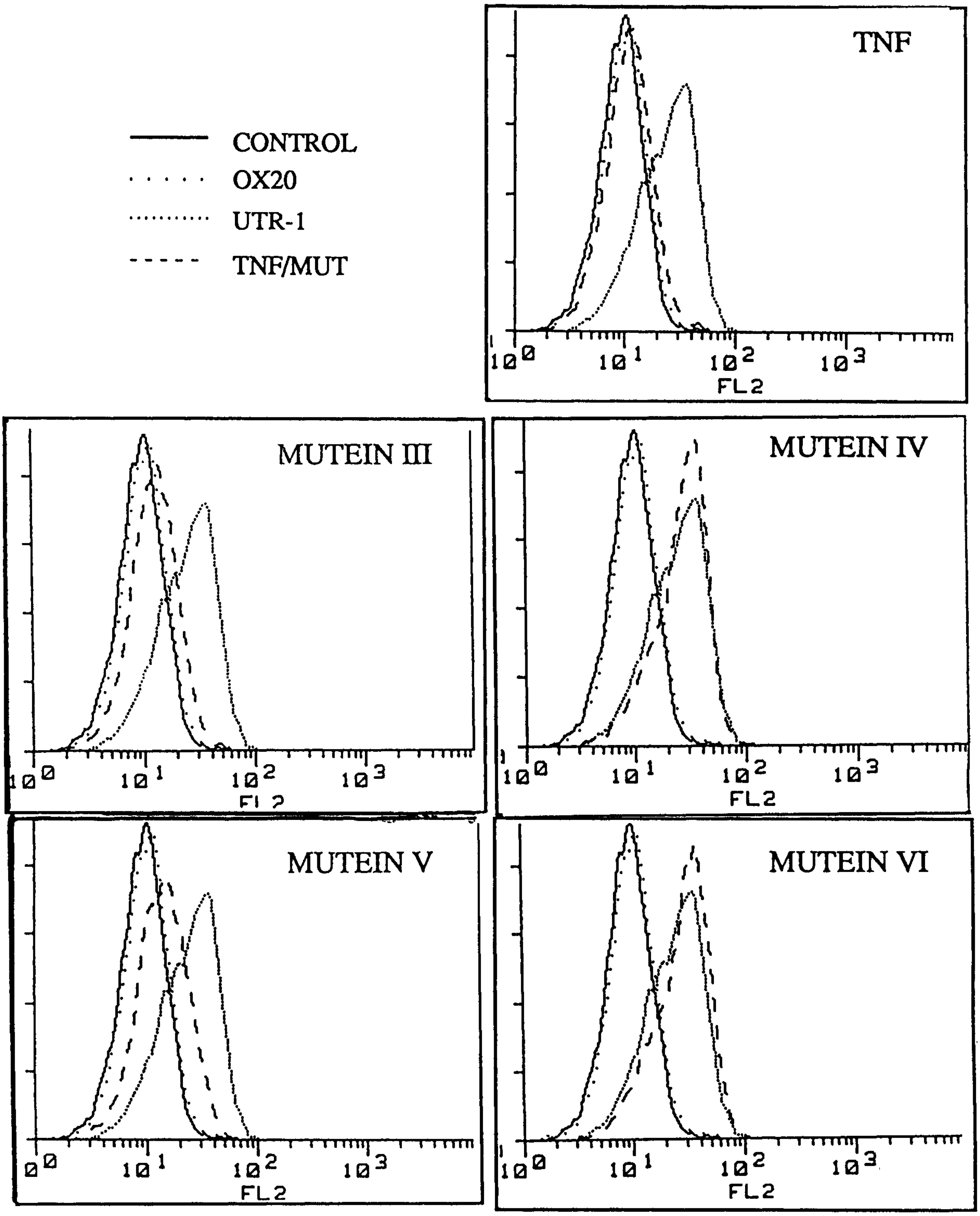

FIG. 2. Flow cytometric analysis of the binding of monoclonal antibodies Utr-1 and OX-20 to human PMN pretreated with TNF-α (100 ng/ml) or muteins III–VI (100 ng/ml) for 30 min at room temperature. TNF-α, mutein III and to a lesser extent mutein V block binding of Utr-1 to PMN.

to addition of fMLP (Figure 4). Pretreatment of PMN with muteins IV and VI failed to enhance fMLP-induced chemiluminescence generation (Figure 4).

*Release of lysosomal enzymes from PMN:* Activation of freshly isolated PMN by a number of agents leads to the release of lysosomal enzymes from neutrophil granules. We have measured the release of two such enzymes—lysozyme and β-glucuronidase—from PMN stimulated with either TNF-α, muteins or fMLP.

Addition of TNF-α, mutein III and mutein V, as well as fMLP, resulted in release of comparable amounts of lysozyme (Table 1). In contrast, muteins IV and VI did not display any activity in this

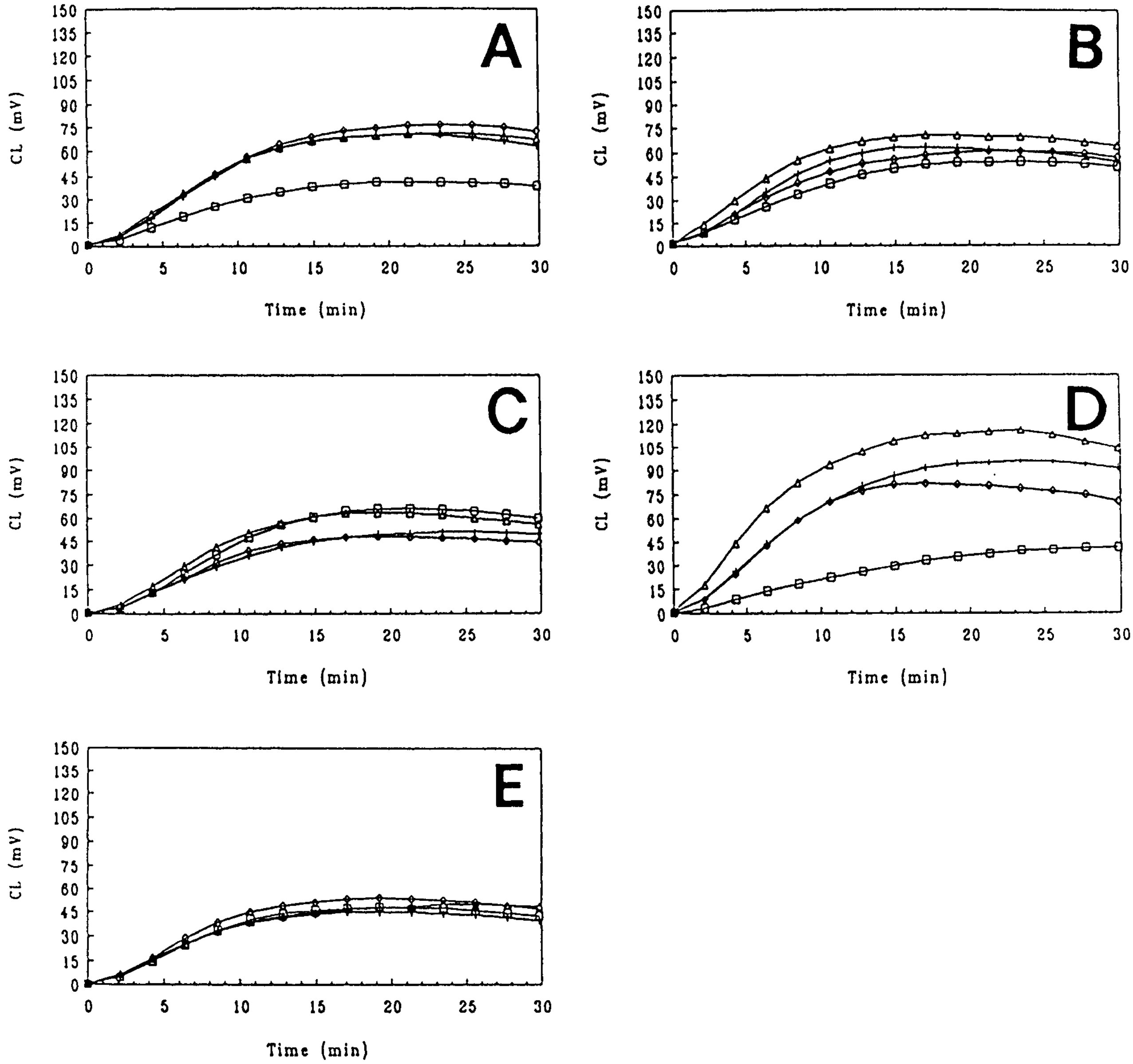

FIG. 3. Chemiluminescence of human PMN stimulated with for 30 min at room temperature with either (A) TNF-α, (B) mutein III, (C) mutein IV, (D) mutein V, (E) mutein VI. CL generation was measured using a Luminometer 1251. TNF-α, mutein III and mutein V, but not muteins IV and VI, stimulate chemiluminescence of human PMN. --□-- Control, --+-- 1 ng/ml, --◆-- 10 ng/ml, --▲-- 100 ng/ml.

system. We have also investigated degranulation of PMN after treatment with cytochalasin B, which affects cytoskeletal proteins. As expected, cytochalasin greatly enhanced fMLP-induced degranulation (Table 1), but was without effect on release of lysozyme in response to either TNF-α or muteins III and V. Interestingly, following cytochalasin B treatment, mutein IV also increased lysozyme release to levels comparable to those observed with the other muteins.

Table 2 shows the results of measurements of β-glucuronidase activity in cell-free extracts. In the absence of cytochalasin B, only fMLP and TNF-α promoted significant release of β-glucuronidase. Addition of cytochalasin B resulted in increased β-glucuronidase activity in supernatants from PMN stimulated with fMLP, TNF-α and mutein III. There was no significant effect on β-glucuronidase activity in supernatants from PMN challenged with muteins IV, V and VI. Statistical analysis of the data revealed that only fMLP-induced release was significantly ($p < 0.05$) above background release.

### Discussion

TNF-α is released during the course of inflammatory reactions by several cell types, chiefly monocytes, but also by PMN,[31] and it participates in the host response to infection and trauma.[1] Moreover TNF-α may cause tissue damage by augmenting PMN function, both by directly activating neutrophils and by affecting their

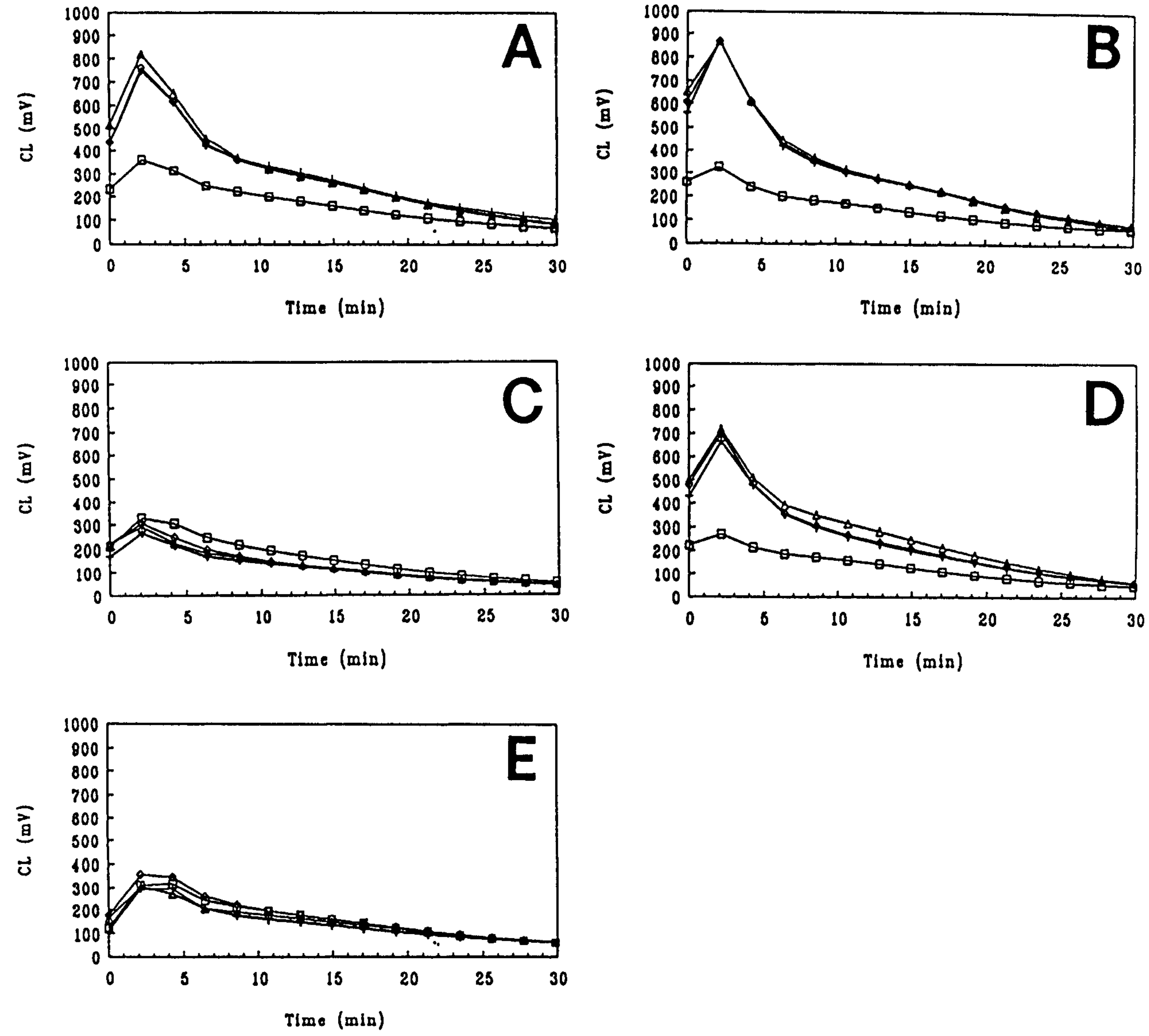

FIG. 4. Chemiluminescence of human PMN in response to fMLP ($10^{-6}$ M) following pretreatment for 30 min at room temperature with either (A) TNF-α, (B) mutein III, (C) mutein IV, (D) mutein V, (E) mutein VI. CL generation was measured using a Luminometer 1251. TNF-α, mutein III and mutein V enhance chemiluminescence in response to fMLP. --□-- Control, --+-- 1 ng/ml, --◆-- 10 ng/ml, --▲-- 100 ng/ml.

**Table 1.** Lysozyme release from PMN exposed to TNF-α and muteins[a]

| Addition | Conc. | Control | Cytochalasin[b] |
|---|---|---|---|
| PBS | | 2.3 ± 0.9 | 2.3 ± 1.0 |
| fMLP | ($10^{-7}$ M) | 25.9 ± 4.4* | 65.7 ± 4.1* |
| TNF-α | (10 ng/ml) | 19.7 ± 8.6* | 21.9 ± 1.1* |
| Mutein III | (10 ng/ml) | 21.8 ± 7.8* | 26.8 ± 2.3* |
| Mutein IV | (10 ng/ml) | 4.4 ± 4.1 | 12.8 ± 1.9* |
| Mutien V | (10 ng/ml) | 17.1 ± 1.4* | 14.1 ± 1.7* |
| Mutien VI | (10 ng/ml) | 4.6 ± 4.4* | 4.8 ± 3.9 |

[a] Enzyme activity (±SD, $n = 4$) in the supernatant was expressed as a percentage of total activity released by 0.2% Triton-X 100. LDH activity was less than 5% of the amount measured in a solution obtained by cell pellet lysis.
[b] PMN ($2 \times 10^6$) were treated with cytochalasin B (5 μg/ml) for 15 min at 37°C, before addition of TNF-α or muteins.
* Statistically significant ($p \leq 0.05$) difference relative to response in the presence of PBS alone determined by Student's *t*-test.

**Table 2.** β-Glucuronidase release from PMN exposed to TNF-α or muteins[a]

| Addition | Conc. | Control | Cytochalasin[b] |
|---|---|---|---|
| PBS | | 3.1 ± 1.4 | 2.9 ± 1.5 |
| fMLP | ($10^{-7}$ M) | 8.1 ± 3.1* | 16.6 ± 1.5* |
| TNF-α | (10 ng/ml) | 8.1 ± 3.6* | 11.0 ± 5.9 |
| Mutein III | (10 ng/ml) | 3.4 ± 2.9* | 14.9 ± 8.5 |
| Mutein IV | (10 ng/ml) | 4.4 ± 4.1 | 7.7 ± 6.7 |
| Mutein V | (10 ng/ml) | 5.7 ± 5.0 | 9.5 ± 6.6 |
| Mutein VI | (10 ng/ml) | 4.8 ± 4.2 | 6.4 ± 5.3 |

[a] Enzyme activity (±SD, $n = 4$) in the supernatant was expressed as a percentage of total activity released by 0.2% Triton-X 100. LDH activity was less than 5% of the amount measured in a solution obtained by cell pellet lysis.
[b] PMN ($2 \times 10^6$) were treated with cytochalasin B (5 μg/ml) for 15 min at 37°C, before addition of TNF-α or muteins.
* Statistically significant ($p \leq 0.05$) difference relative to response in the presence of PBS alone determined by Student's *t*-test.

responses to other stimuli such as fMLP. For example, TNF-α enhances phagocytosis, oxidative metabolism, aggregation, degranulation and adherence of PMN following binding to its specific cell surface receptors.[12–15] Its effects on PMN function can be completely blocked by anti-TNF-α antibodies and TNF-α inhibitors.[32,33]

In the present study, the effects of TNF-α derivatives termed muteins, which differ from native TNF-α in their amino acid composition, on human PMN were investigated in order to examine the structural requirements of TNF-α for binding to PMN receptors. Since antibodies recognizing the N-terminus of TNF-α block its attachment to cellular receptors and inhibit biological effects of TNF-α[21,22] we have replaced the 3–7 N-terminal amino acids of TNF-α, yielding four muteins with hydrophobic/hydrophilic characteristics different to those of the native molecule. The results confirm earlier observations[20] that human PMN appear to express predominantly the 75 kDa TNF receptor type, as shown by the flow cytometric analysis of the binding of monoclonal antibodies against the p75 TNF receptor. In contrast, there was no detectable binding of anti-p55 TNF receptor antibodies to PMN. Binding to p75 was abolished by addition of TNF-α, and muteins III and V, but not muteins IV and VI. In an earlier study the authors compared the ability of TNF-α and muteins to compete with anti-TNF receptor antibodies for binding to Jijoye Burkitt lymphoma cells and to the human epithelioid carcinoma HeLa cell line, which selectively express either p75 or p55 receptors alone. The authors demonstrated that TNF-α, mutein III and mutein V bind to HeLa cells, whereas only TNF-α and mutein III competed with anti-p75 antibodies for binding to the p75 receptor on Jijoye cells. Muteins IV and VI failed to recognize either TNF receptor species.[23] The ability of mutein III, therefore, to prevent anti-p75 monoclonal antibody binding to PMN further supports the observations that the surface receptor on PMN for TNF-α is primarily the 75 kDa sub-type. This is also corroborated by the data showing enhancement by mutein III of superoxide anion production by PMN in response to fMLP. Finally, mutein III, like native TNF-α itself, induces release of lysozyme from PMN, presumably as a result of binding to the p75 receptor. Secretion of β-glucuronidase in response to mutein III was only observed following cytochalasin B treatment of PMN, although this release was not apparently significantly above background levels.

Since mutein V did not affect binding of anti-p75 to Jijoye cells,[23] its activity in inhibiting anti-p75 receptor binding to PMN appears inconsistent with the preponderance of p75 on PMN. It is possible that this mutein binds to a different epitope on Jijoye p75 to that recognized by Utr-1, and thus appears not to affect binding of this antibody to Jijoye cells. Mutein V also induced neutrophil responses comparable to those elicited by TNF-α and mutein III, despite the apparent absence of p55 on PMN. However, Porteu and colleagues[18] clearly demonstrated inhibition by anti-p55 antibody Htr-9 of $^{125}$I-TNF-α binding to PMN, suggesting that p55 receptors are indeed present on these cells. It was also reported by the same group that activated human PMN release soluble fragments of both TNF receptors. This shedding of cell surface receptors occurs by at least two distinct mechanisms. Exposure of PMN to sonicated neutrophil azurophil granules results in almost exclusive release of a fragment of the p75 TNF receptor, by an elastase dependent mechanism. In contrast, stimulation of PMN by fMLP leads to shedding of similar amounts of both p55 and p75, and is insensitive to elastase inhibitors.[18] It is therefore clear that p55 is indeed expressed on the surface of human PMN. In the flow cytometric studies, it is possible that although binding of anti-p55 antibodies to PMN was not detectable, low levels of p55 are sufficient to allow binding to this receptor and hence initiation of neutrophil responses by mutein V.

Muteins IV and VI did not compete with anti-TNF receptor antibodies for binding to PMN, which is in accordance with their lack of ability to block binding of Utr-1 and Htr-9 to Jijoye and HeLa cells.[23] In addition, these compounds did not induce superoxide anion production, either alone, or in combination with fMLP, and were without effect on secretion of β-glucuronidase. Lysozyme release from PMN in the absence of cytochalasin B was also unaltered, although in the presence of cytochalasin B mutein IV induced significant release of this enzyme into the cell supernatant. The mechanism responsible for this effect is at present unclear.

The results presented in this report confirm that N-terminal amino acids are critical for both binding to neutrophils and induction of PMN responses by TNF-α. This is in agreement with previously published data showing that changes in the basicity of N-terminal amino acids affect the cytotoxic activity of TNF-α,[34] as well as with our own findings of differential binding of muteins to HeLa and Jijoye cells.[23] The question of structure–function relationships to TNF-α activity has also been addressed by other investigators.[35–37] For example, a histidine residue at position 15 of TNF-α was found to be an essential requirement for cytotoxic activity,[35] whereas in another study chemical modifications of N-terminal amino groups revealed a strong correlation between the extent of modification and biological activity.[36] Details in this report also describe that the two muteins with hydrophilic N-terminal portions similar to those of native TNF-α are effective in inducing neutrophil responses by binding to cell surface receptors. In contrast, hydrophobic muteins IV and VI failed to activate neutrophils in our system. In summary, it appears that recognition of receptors on PMN and induction of cellular responses by TNF-α is governed at least in part by the nature of the N-terminal amino acids and the hydrophilic properties of this pleiotropic cytokine.

### References

1. Sherry B, Cerami A. Cachectin/tumour necrosis factor exerts endocrine, paracrine and autocrine control of inflammatory responses. *J Cell Biol* 1988; **107**: 1269–1277.
2. Ziegler EJ. Tumour necrosis factor in humans. *New Engl J Med* 1988; **318**: 1533–1535.
3. Beutler B, Cerami A. Cachetin: more than a tumour necrosis factor. *New Engl J Med* 1990; **316**: 379–385.
4. Hohmann HP, Remy R, Brockhaus M, van Loon AP. Two different cell types have different major receptors for human tumour necrosis factor. *J Biol Chem* 1989; **264**: 14927–14934.
5. Brockhaus M, Schoenfeld HJ, Schlager EJ, Hunziker W, Lesslauer W, Loetscher H. Identification of two types of tumour necrosis factor receptors on human cell lines by monoclonal antibodies. *Proc Natl Acad Sci USA* 1990; **87**: 3127–3131.
6. Gray PW, Barrett K, Chantry DH, Turner M, Feldmann M. Cloning of human tumour necrosis factor (TNF) receptor cDNA and expression of recombinant soluble TNF-binding protein. *Proc Natl Acad Sci USA* 1990; **87**: 7380–7384.
7. Schall TJ, Lewis M, Koller KJ, *et al.* Molecular cloning of a receptor for human tumour necrosis factor. *Cell* 1990; **61**: 361–370.
8. Smith CA, Davis T, Anderson D, *et al.* A receptor for tumour necrosis factor defines an unusual family of cellular and viral proteins. *Science* 1990; **248**: 1019–1023.
9. Espevik T, Brockhaus M, Loetscher H, Nonstad U, Shalaby R. Characterization of binding and biological effects of monoclonal antibodies against a human tumour necrosis factor receptor. *J Exp Med* 1990; **171**: 415–426.
10. Ryffel B, Brockhaus M, Greiner B, Mihatsch MJ, Gudat BM. Tumour necrosis factor distribution in human lymphoid tissue. *Immunology* 1991; **74**: 446–452.
11. Tartaglia LA, Weber RF, Figari IS, Reynolds C, Palladino MA, Goeddel DV. The two different receptors for tumour necrosis factor mediate distinct cellular responses. *Proc Natl Acad Sci USA* 1991; **88**: 9292–9296.
12. Larrick JW, Graham D, Toy K, Lin LS, Senyk G, Fendly BM. Recombinant tumour necrosis factor causes activation of human granulocytes. *Blood* 1987; **69**: 640–644.
13. Steinbeck MJ, Roth JA. Neutrophil activation by recombinant cytokines. *Rev Infect Dis* 1989; **11**: 549–568.
14. Arnaout MA. Leukocyte adhesion molecule deficiency: its structural basis, pathophysiology and implications for modulating the inflammatory response. *Immunol Rev* 1990; **114**: 146–180.
15. Limb GA, Hamblin AS, Wolstencroft RA, Dumonde DC. Selective upregulation of human granulocyte integrins and complement receptor 1 by cytokines. *Immunology* 1991; **74**: 696–702.
16. Shalaby MR, Palladino MA, Hirabayashi SE, *et al.* Receptor binding and activation of polymorphonuclear neutrophils by tumour necrosis factor-α. *J Leukocyte Biol* 1987; **41**: 196–204.
17. Pichyangkul S, Schick D, Jia F, Berent S, Bollon A, Kahn A. Binding of tumour necrosis factor-alpha (TNF-α) to high-affinity receptors on polymorphonuclear cells. *Exp Hematol* 1987; **15**: 1055–1059.
18. Porteu F, Brockhaus M, Wallach D, Engelmann H, Nathan C. Human neutrophil elastase releases a ligand-binding fragment from the 75-kDa tumour necrosis factor (TNF) receptor. *J Biol Chem* 1991; **266**: 18846–18853.
19. Porteu F, Nathan C. Shedding of tumour necrosis factor receptors by activated human neutrophils. *J Exp Med* 1990; **172**: 599–607.
20. Zeman K, Tchorzewski H, Paleolog E, Brennan F, Feldmann M. Identification of TNF receptors on human polymorphonuclear neutrophils by monoclonal antibodies. 1992; Submitted for publication.
21. Socher SH, Riemen MW, Martinez D, *et al.* Antibodies against amino acids 1–15 of tumour necrosis factor block its binding to cell-surface receptor. *Proc Natl Acad Sci USA* 1987; **84**: 8829–8833.
22. Goh CR, Porter AG. Structural and functional domains in human tumour necrosis factors. *Protein Eng* 1991; **4**: 386–389.
23. Tchorzewski H, Zeman K, Paleolog E, *et al.* The effects of tumour necrosis factor (TNF) derivatives on TNF receptors. 1992; Submitted for publication.
24. Klysik J, Konarzewska-Zglinska A, Galazka G, *et al.* Synthesis and expression in *E. coli* of the gene for tumor necrosis factor. *Arch Immunol Therap Exp* 1992; **39**: 349–354.
25. Zeman K, Tchorzewski H, Majewska E, Pokoca L, Pinkowski R. A simple and rapid method for simultaneous purification of peripheral blood lymphocytes and granulocytes. *Immunol Pol* 1988; **13**: 217–224.
26. Cope AP, Aderka D, Doherty M, *et al.* Soluble tumour necrosis factor (TNF) receptors are increased in the sera and synovial fluids of patients with rheumatic diseases. *Arthritis Rheum* 1992; **35**: 1100–1109.
27. Ghebrehiwet B. The release of lysosomal enzymes from human polymorphonuclear leukocytes by human C3a. *Clin Immunol Immunpathol* 1984; **30**: 321–329.
28. Wright DG. Human neutrophil degranulation. *Methods Enzymol* 1988; **162**: 538–551.
29. Kantorski J, Tchorzewski H. The effects of serine and thiol inhibitors on the chemiluminescence of human neutrophils in investigations *in vitro*. *J Biolum Chemilum* 1992; **7**: 37–45.
30. Kyte J, Doolittle RF. A simple method for displaying the hydrophobic character of a protein. *J Mol Biol* 1982; **157**: 105–132.
31. Dubravec DB, Spriggs DR, Mannick JA, Rodrick ML. Circulating human peripheral blood granulocytes synthesise and secrete tumour necrosis factor-α. *Proc Natl Acad Sci USA* 1990; **87**: 6758–6761.
32. Ferrante A, Hauptmann B, Seckinger P, Dayer JP. Inhibition of tumour necrosis factor alpha (TNF-α)-induced neutrophil respiratory burst by a TNF inhibitor. *Immunology* 1991; **72**: 440–442.
33. Cerami A. Inflammatory cytokines. *Clin Immunol Immunopathol* 1992; **62**: S3–S10.
34. Soma GI, Kitahara N, Tsuji Y, *et al.* Improvement of cytotoxicity of tumour necrosis factor (TNF) by increase in the basicity of its N-terminal region. *Biochem Biophys Res Commun* 1987; **148**: 629–635.
35. Yamamoto R, Wang A, Vitt CR, Lin LS. Histidine-15: an important role in the cytotoxic activity of human tumour necrosis factor. *Protein Eng* 1989; **2**: 553–558.
36. Utsuni T, Hung MC, Klostergaard J. The role of amino functions in recombinant human tumour necrosis factor in expression of biological activity. *Molec Immunol* 1992; **29**: 77–81.
37. Yamagushi J, Kawashima H, Matsuo N. Mutational analysis of structure–activity relationships in human tumor necrosis factor-alpha. *Protein Eng* 1990; **3**: 713–719.

ACKNOWLEDGEMENTS. This work was supported by grant No. 40209101 from the Committee for Research and Science, Poland, and from the Polish Science Foundation, by the Arthritis and Rheumatism Council and British Heart Foundation (EMP), and by a British Council Academic Link Agreement. The authors thank Prof. W. Stec for providing recombinant human TNF-α.

**Received 10 November 1992; accepted 1 December 1992**
